# The association between systolic and diastolic dysfunction and autonomic nervous system function in children receiving chronic hemodialysis

**DOI:** 10.1007/s00467-024-06577-1

**Published:** 2025-01-28

**Authors:** Ola H. Abd Elaziz, Ghada M. S. Ahmad, Salwa S. Abd Elgawad, Fatma Elhady, Rehab M. Hamdy

**Affiliations:** 1 Cardiology Department, Faculty of Medicine (Girls), Al-Azhar University, Cairo, Egypt; 2 Pediatric Department, Faculty of Medicine (Girls), Al-Azhar University, Cairo, Egypt

**Keywords:** Chronic kidney disease, Speckle tracking echocardiography, Heart rate variability, Global longitudinal shortening

## Abstract

**Background:**

Changes in cardiac function and structure as well as their association with the cardiac autonomic nervous system remain incompletely characterized in children with stage 5 chronic kidney disease (CKD) receiving hemodialysis (HD).

**Methods:**

A prospective observational cohort study was conducted on 40 Egyptian children with CKD on regular HD compared to 40 age- and sex-matched healthy children. All participants underwent thorough clinical examination, laboratory investigations, 24-h Holter monitoring, and 2D/4D echocardiographic study (conventional and advanced modalities). Participants were followed for mortality and morbidity over 36 months.

**Results:**

Following HD sessions, CKD children showed significant reductions of left and right ventricular (LV/RV) systolic function by 2D and 4D echocardiography compared to controls. HD children had significant impairment of heart rate variability parameters (evaluated by time and frequency domains). LV/RV global longitudinal shortening (GLS) as well as tricuspid annular plane systolic excursion were closely correlated with different Holter parameters, including frequency domain parameters (including low frequency, high frequency, and LF/HF ratio), time domain parameters including percentage of differences > 50 ms between consecutive normal RR intervals (pNN50), and root-mean-square of the difference between successive normal intervals (rMSSD). Over a follow-up of 34.5 ± 16.8 months, 10 (25%) patients died. Reduced LV/RV-GLS and decreased rMSSD values were independently associated with higher mortality among HD children.

**Conclusions:**

LV and RV myocardial deformation (either 2D or 4D) primarily decreased in HD children. Altered time and frequency domain indices revealed cardiac autonomic dysfunction, evidenced by increased sympathetic activity and decreased vagal activity. Reduced LV/RV-GLS and decreased rMSSD values were independently associated with higher mortality among HD children.

**Graphical abstract:**

**Figure:**
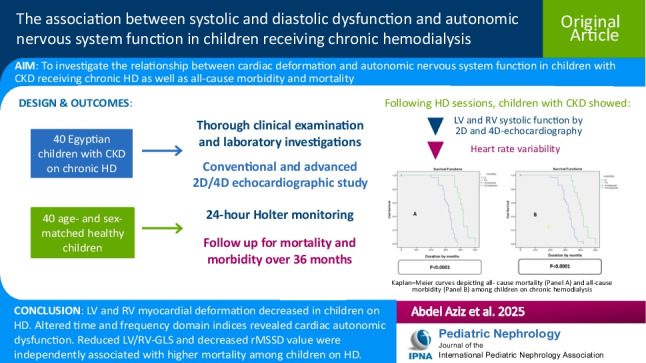
A higher resolution version of the Graphical abstract is available as A higher resolution version of the Graphical abstract is available as Supplementary information

**Supplementary Information:**

The online version contains supplementary material available at 10.1007/s00467-024-06577-1.

## Introduction

With up to 30% of pediatric hemodialysis (HD) mortality attributed to cardiovascular events, cardiovascular complications are common in children with stage 5 chronic kidney disease (CKD) [1]. Even if ejection fraction (EF) is still preserved, advanced echocardiography methods to assess myocardial performance may be valuable because the deterioration of left ventricular (LV) function is a primary determinant of survival in patients on HD [2]. For the assessment of LV size and function, four-dimensional (4D) echocardiography provides greater accuracy and reproducibility than two-dimensional (2D) echocardiography [3]. This is because 4D echocardiography avoids apical foreshortening and is based on direct volumetric measurements without geometrical assumptions. 4D echo-derived global longitudinal shortening (GLS) may be more precise and consistent since it is capable of tracking myocardial motion regardless of the imaging plane [4]. Furthermore, 4D EF is a better predictor of cardiovascular mortality than 2D EF, and it is beneficial to include GLS, which better predicts survival, in addition to other known risk variables [5].

LV systolic function is impaired in young adults with CKD even in the early stages [6]. 2D echocardiography and tissue Doppler measures have been used in observational studies to characterize the general preservation of systolic LV function. Early alterations in cardiac mechanics that occur before changes in the EF have been described by speckle tracking echocardiography (STE). A reduction in strain values has been linked to a decline in kidney function in adults with CKD [7, 8]. Conversely, a decrease in longitudinal shortening has been linked to a substantial risk factor for all-cause mortality in patients with CKD [9].

A new and more precise method for evaluating regional and global myocardial functions is cardiac deformation imaging. Deformation imaging depends on the distinct orientation of cardiac fibers and their mechanical properties [10]. The assessment of cardiac deformation is possible with STE [11]. STE has been found to play a role in the early diagnosis of cardiac involvement by assessing myocardial deformation in three myocardial layers: longitudinal shortening, circumferential shortening, and radial shortening [12]. Prior to the onset of EF abnormalities, early changes in myocardial mechanics can be identified by STE [13]. LV-GLS using 2D-STE is a predictor of sudden cardiac death (SCD) and ventricular arrhythmia in adult dialysis patients [14]. There is limited data available about shortening imaging and 4D echocardiographic ventricular function as indicators of ventricular dysfunction in children with CKD on regular HD [15].

The autonomic nervous system (ANS), which balances the involvement of both the sympathetic and parasympathetic nervous systems, controls the cardiovascular system by regulating heart rate, conduction velocity, and contraction force. The influence of the ANS on heart rate variability (HRV) is reflected in the sympathovagal balance; alterations to this balance lead to autonomic dysfunction [16]. Hemodialysis patients have been reported to experience cardiac involvement and ANS dysfunction. There have been reports of abnormal circadian blood pressure patterns and decreased baroreceptor sensitivity; further studies suggest that ANS dysfunction may raise the risk of arrhythmias and unexpected deaths [17]. Our objective was to investigate the association between left and right ventricular deformation identified by STE and ANS function indices (both time and frequency domain) in children with CKD receiving chronic regular HD. Additionally, all-cause morbidity and mortality were assessed in children on chronic HD.

## Patients and methods

### Study design and setting

This is a prospective observational cohort study conducted on 40 consecutive Egyptian pediatric patients with CKD on dialysis visiting the pediatric nephrology unit at Al Azhar University Hospital and 40 healthy age- and sex-matched controls. The study was approved by the Ethical Committee of the Faculty of Medicine at Al Azhar University with approval number 2018122001. Written informed consent was obtained from all participants’ parents or legal guardians. Additional informed consent was obtained from all individual participants for whom identifying information is included in this article.

### Definitions

#### Chronic HD

We recruited only children with stage 5 CKD according to the Kidney Disease Improving Global Outcomes (KDIGO) staging scale [18] who were on HD protocol (for at least 6 months) consisting of 3 sessions per week, 4 h each, who were eligible to participate in the study.

#### Global longitudinal shortening (GLS)

Myocardial shortening, expressed as a percentage, is a dimensionless measure of the overall ventricular myocardial deformation during a cardiac cycle [19]. The fractional change in the length of a portion of the myocardium relative to its initial length is calculated using ultrasonic wave frequency shifts. With STE, strain and deformation of the heart are measured by tracking the natural acoustic reflectors, or “speckles,” in the heart from frame to frame. At a frame rate of between 70 and 90 frames per second, 2D grayscale images were acquired in the apical chamber views. Three heart cycles were recorded. A workstation was used for offline analysis. At the end of systole, the endocardial border was manually drawn. The software should also suggest sufficient tracking. The tracking was completed automatically, and the analysis was validated following a thorough check. From the 4-, 2-, and 3-chamber views of the LV and the 4-chamber view of the RV, the apical, mid, and basal segments were obtained [13].

#### Tricuspid annular plane systolic excursion (TAPSE)

We placed the cursor on the lateral tricuspid annulus close to the free RV wall, aligning it as closely as possible to the heart’s apex. We quantified TAPSE in two-dimensional M-mode echocardiograms from the 4-chamber view.

Heart rate variability (HRV) parameters were assessed using 24-h ambulatory ECG (Holter) monitoring after the HD session. The following time and frequency domain parameters were measured [20]:

1. Mean RR (ms): the mean of the RR interval.
2. pNN50 (%): the percentage of differences > 50 ms between successive normal RR intervals in a 24-h ECG record. It predominantly reflects parasympathetic activity.
3. rMSSD (ms): the root mean square of the difference between successive normal intervals. It is an important indicator of parasympathetic activity.
4. LF (nu): it includes the absolute power of the low-frequency band range between 0.04 and 0.15 Hz and consists of a combination of sympathetic and parasympathetic effects.
5. HF (nu): it includes the absolute power of the high-frequency band range between 0.16 and 0.4 Hz. It is considered to be modulated by the parasympathetic activity of the ANS.
6. LF/HF ratio: the ratio of LF-to-HF power. It reflects the sympathovagal balance and can be used to estimate HRV in general.

#### Follow-up and outcomes

Patients were followed for the primary endpoint (all-cause mortality) and secondary endpoints (all-cause morbidity encompassing recurrent infections defined as access-related bloodstream infections or blood-borne virus infections, particularly hepatitis C and B or respiratory tract infections, significant arrhythmias defined as non-fatal and fatal arrhythmias, including atrial fibrillation and ventricular arrhythmia, cerebrovascular stroke either ischemic or hemorrhagic, and complications associated with vascular access defined as infections that can lead to hospitalization or access failure). The outcomes were obtained from documented medical records for each patient.

### Case search strategy and study population

Eighty-five children were recruited. Eighty children from the target population whose files comprise all unit attendants met the selection criteria. Five children declined to continue their participation during the follow-up. All patients were in sinus rhythm with an absence of primary myocardial disease. Children with stage 5 CKD according to the KDIGO staging scale [18] who were on HD protocol for at least 6 months in the form of 3 sessions per week, 4 h each, were eligible to participate in the study. Forty healthy children from the same geographic area, socioeconomic status, and ethnic group matched to the patients’ group were included as the control group. They were matched for age and sex. They were collected from the outpatient pediatric clinic for periodic exams and evaluation. A physical examination and medical history were used to evaluate each child’s health.

### Exclusion criteria

The presence of non-sinus rhythm, myocardial or pericardial disease, severe valvular heart disease, or congenital heart disease excluded participants from the study.

### Characterization of cases

Forty Egyptian children with stage 5 CKD on regular HD were recruited for the study. Fourteen of them were female, and their ages were 12.3 ± 2.8 years. Seven cases were aged between 6 and 9 years, 24 cases were aged between 10 and 14 years, and 9 cases were aged between 15 and 17 years.

### History taking and clinical examination

All studied cases underwent thorough history-taking with special emphasis on the onset and etiology of kidney disease, duration of HD, and their medications (such as anti-hypertensive drugs or immunosuppressive treatment). A comprehensive assessment included recording vital signs, body weight, dry weight, height, body surface area (BSA), body mass index (BMI), heart rate (HR), and post-dialysis blood pressure (BP) measurements and BP percentile.

Laboratory investigations were conducted before the midweek dialysis session and included a complete blood count (CBC), blood urea, serum creatinine, serum sodium (Na), potassium (K), calcium (Ca), magnesium (Mg), and phosphate (Ph).

24-h ambulatory ECG (Holter) monitoring was performed, and the heart rate variability (HRV) parameters were assessed using 24-h ambulatory ECG (Holter) monitoring after the HD session and 24 h after stopping antihypertensive medications. Data collection and processing were done using Windows 7 software in conjunction with Holter (Cardiomera, Meditech, Hungary). The 3-channel ECG was analyzed for the entire day. Two different time periods were observed for the HRV parameters: overnight (12:00–8:00 a.m.) and daytime (8:00 a.m.–12:00 a.m.). Time and frequency domain parameters were evaluated in all participants.

A conventional transthoracic echo-Doppler study was performed 60 min following the HD session, and all images were taken after resting during quiet respiration in the left lateral decubitus position. All cases were subjected to a conventional transthoracic echo-Doppler study using the Vivid-E9 GE system (GE Ultrasound; Horten, Norway) with a multi-frequency (2.5 MHz) matrix probe M3S parallel ECG physio-recording signals displayed with all recorded echocardiographic images and loops. GE version 110–1.3 of EchoPAC was used for an offline analysis. All parameters were acquired in accordance with the American Society of Echocardiography guidelines [21]. Measurements were performed regarding LV volumes, EF, LV mass, LV mass indexed to the body surface area and height (LVMI), E and A velocities, and the E/A ratio for the flow through the mitral and tricuspid valves. Mitral and tricuspid annular systolic and diastolic velocities were measured using tissue Doppler imaging (TDI) in addition to the calculation of the E/E′ ratio. Mitral annulus plane systolic excursion (MAPSE), TAPSE, and both LV and RV myocardial performance indices (LV-MPI, RV-MPI) were obtained [21].

2D STE was used for the assessment of myocardial deformation, LV-GLS, and RV-GLS. The cutoff values that we employed were 20.6% for LV-GLS and 28.2% for RV-GLS [22].

4D echocardiography was performed to assess LV volumes, functions, and shortening in addition to RV volumes, functions, TAPSE, and fractional area change (FAC).

### Follow-up and outcomes

Patients were followed for a duration of 36 months with respect to the following endpoints: *the primary endpoint was all-cause mortality,* while the secondary endpoint was all-cause morbidity.

### Statistical analysis

#### Sample size and sampling

After calculating the sample size with a 95% significance level, 80% power, and 30% effect size, 85 children were recruited. Eighty children from the target population, whose files comprise all unit attendants, were included. Five children declined to continue participation during the follow-up.

The software SPSS 23.0 (SPSS Inc., Chicago, IL, USA) was used for data analysis. The Shapiro–Wilk test was used to assess the distribution of the data. Continuous data were expressed as means ± SD. Variables between groups were compared using Student’s unpaired *t*-test, chi-square analysis, and univariate analysis. Pearson and Spearman correlation analyses were performed to assess the association between variables. Analysis of the Receiver Operating Characteristics (ROC) curve was done to determine the cutoff value of 4D LV-EF, 4D LV-GLS, 4D TAPSE, 4D RV-EF, and 4D FAC for the detection of systolic dysfunction among HD children. A Kaplan–Meier curve was developed to compare the event-free survival rate. For the mortality analysis, the log-rank test was also employed. Based on the patient’s first documented dialysis treatment date, the study’s starting point was determined to be the time of HD. Therefore, the outcome variable was measured from the time HD started until death. Right-censoring was applied to patients who had partial failure to follow up, some HD sessions in another center, or remained at the end of the study. In this study, censoring was considered to be either independent or non-informative (with the percentage of censored cases being 50%). A univariate Cox regression analysis was conducted with all the variables included. A statistically significant *P*-value is < 0.05.

## Results

The study population was classified into two groups. Group I included 40 children with CKD (stage 5 CKD) on regular HD. Group II included 40 healthy children matched for age and sex.

**Fig. 1:**
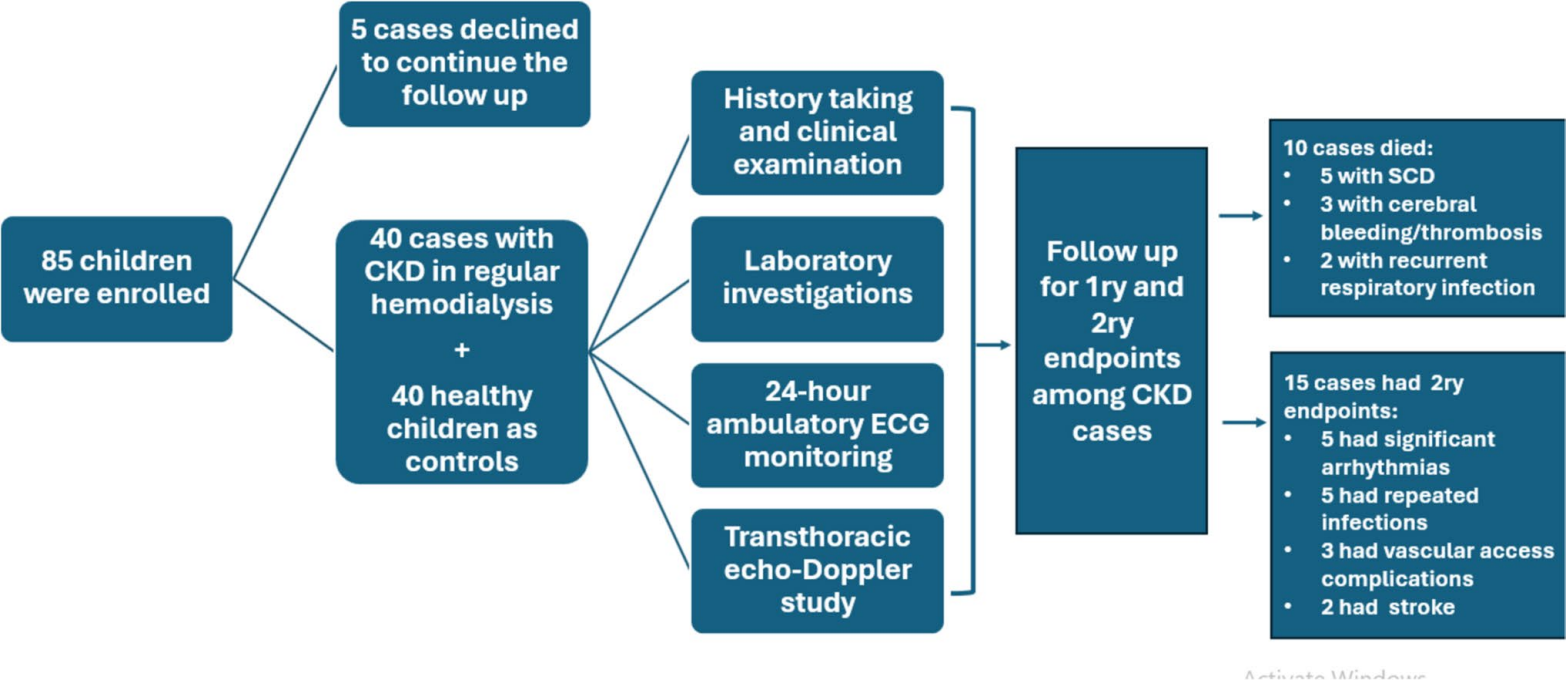
Flowchart for the enrolled children with stage 5 chronic kidney disease and controls in the current study. CKD, chronic kidney disease; ECG, electrocardiography; SCD, sudden cardiac death

Figure 1 summarizes the flow chart for the enrolled CKD children and controls.

Table 1 provides the demographic information and baseline laboratory findings for the study population. Table 2 shows the etiologies of CKD in the included patients. The duration of HD in children with CKD was 32.5 ± 6.5 months with an average amount of fluid removal per session of 2.3 ± 1.5 kg. There were no significant differences between children on HD and controls regarding age, sex, weight, BSA, and BMI. However, children on HD had significantly higher post-dialysis systolic and diastolic BP, white blood cells (WBCs), Ph, Mg, and K compared to the control group. On the other hand, children on HD had significantly lower RBCs, Hb%, platelets, and Ca compared to the control group.

**Table 1:** Study population characteristics including demographic, clinical data, and laboratory investigations

Variables	Cases (*N* = 40)	Controls (*N* = 40)	*P*
Age (ys) (mean ± SD)	12.3 ± 2.8	11.6 ± 2.2	0.222
Age groups (number, %)			
• 6 to 9 (ys)	7 (17.5%)	8 (20%)	
• 10 to 14 (ys)	24 (60%)	26 (65%)	
• 15 to 17 (ys)	9 (22.5%)	6 (15%)	
Sex (F/M)	14/26	7/13	0.99
Weight (Kg)	28.3 ± 11.1	27.6 ± 5.0	0.693
Dry weight (Kg)	26.0 ± 10.7	27.6 ± 5.0	0.01
Height (cm)	118.0 ± 11.7	118.5 ± 8.6	0.06
BSA (m^2^)	1.0 ± 0.2	1.1 ± 0.2	0.107
BMI (kg/m^2^)	18.2 ± 4.1	19.7 ± 3.3	0.083
Post-dialysis SBP (mmHg)	130.3 ± 14.6	110.3 ± 9.1	0.0001
Post-dialysis DBP (mmHg)	82.5 ± 13.5	68.5 ± 8.3	0.01
SBP percentile	95.5 ± 8.2	86.8 ± 16.5	0.0001
DPB percentile	88.6 ± 17.3	75.7 ± 21.1	0.01
WBCs (× 10^3^/cmm)	7.5 ± 2.8	5.7 ± 1.1	0.0001
RBCs cell/mcL	3.5 ± 0.8	4.2 ± 0.4	0.0001
HB (gm/dL)	9.0 ± 1.4	11.9 ± 1.2	0.0001
Platelets (× 10^3^/cmm)	242.1 ± 107.2	302.3 ± 53.9	0.002
Urea (mg/dL)	159.8 ± 50.0	21.3 ± 6.2	0.0001
Creatinine (mg/dL)	7.5 ± 2.9	0.8 ± 0.2	0.0001
Ca (mg/dL)	9.1 ± 1.5	10.3 ± 1.0	0.002
Ph (mg/dL)	5.8 ± 1.8	3.4 ± 0.6	0.0001
Mg (mg/dL)	2.8 ± 0.5	1.9 ± 0.2	0.0001
Na (mEq/L)	135.5 ± 2.5	139.3 ± 2.2	0.0001
K (mEq/L)	5.6 ± 1.3	4.1 ± 0.3	0.0001
Current medications:			
• ACEI/ARBs	18 (45%)		
• BB	16 (40%)		
• CCB	14 (35%)		

*BSA* body surface area, *BMI* body mass index, *SBP* systolic blood pressure, *DBP* diastolic blood pressure, *BP* blood pressure, *ACEI* angiotensin-converting enzyme inhibitor, *ARB* angiotensin receptor blocker, *BB* beta blocker, *CCB* calcium channel blocker

**Table 2:** Etiologies of CKD in study subjects receiving hemodialysis

Etiologies of stage 5 CKD:	
• Focal segmental glomerular sclerosis	11 (27.5%)
• Vesico-ureteral reflux	8 (20%)
• Posterior urethral valve with reflux	4 (10%)
• Bilateral atrophic kidneys	4 (10%)
• Nephronophthisis	3 (7.5%)
• Nephrotic syndrome	2 (5%)
• Lupus nephritis	2 (5%)
• Bartter syndrome	2 (5%)
• Bardet-Biedl syndrome	1 (2.5%)
• Autosomal recessive polycystic kidney disease	1 (2.5%)
• ANCA glomerulonephritis	1 (2.5%)
• C3 glomerulopathy	1 (2.5%)

*CKD* chronic kidney disease, *ANCA* anti-neutrophil cytoplasmic antibody

After the HD sessions, Table 3 shows the 24-h Holter monitoring indices. Children undergoing HD had significantly reduced frequency indices as evidenced by impaired low and high frequency and impaired parasympathetic activity detected by low pNN50, rMSSD, and SDNN compared to healthy children. Additionally, the LF/HF ratio and heart rate (minimum and average) of the children on HD were significantly greater than in controls. Furthermore, arrhythmias such as premature ventricular contractions (PVCs) and premature atrial contractions (PACs) were observed in children on HD.

**Table 3:** 24-Hour Holter indices in children receiving chronic hemodialysis and controls

Variables	Cases (*N* = 40)	Controls (*N* = 40)	*P*
HR max (bpm)	171.4 ± 10.2	166.4 ± 8.9	0.066
HR min (bpm)	68.0 ± 10.5	55.0 ± 4.5	0.0001
HR Av (bpm)	100.2 ± 10.4	76.6 ± 7.4	0.0001
LF (nu)	33.3 ± 5.8	69.5 ± 11.9	0.0001
HF (nu)	16.8 ± 3.0	72.6 ± 13.1	0.0001
LF/HF	2.0 ± 0.4	1.0 ± 0.1	0.0001
pNN50 (%)	4.9 ± 3.6	16.5 ± 3.4	0.0001
rMSSD (ms)	22.7 ± 4.6	48.6 ± 7.6	0.0001
SDNN (ms)	66.5 ± 15.0	134.0 ± 14.6	0.0001
Arrhythmias			
• PACs	10 (25%)	-	
• PVCs	5 (12.5%)	-	

*HR* heart rate, *max* maximum, *min* minimum, *Av* average, *LF* low frequency, *HF* high frequency, *nu* normalized unit, *pNN50* percentage of differences > 50 ms between successive normal RR intervals, *rMSSD* root mean square of the difference between successive normal intervals, *SDNN* standard deviation of the RR interval, *PACs* premature atrial contractions, *PVCs* premature ventricular contractions

Regarding 2D/4D echocardiography after HD sessions, the parameters are listed in Tables 4 and 5. In comparison to controls, children on HD had significantly lower LV and RV systolic function parameters (identified by impaired LV/RV myocardial performance index, EF-MM/2D, MAPSE, LV systolic velocity by TDI, LV/RV-GLS, TASPE, and RV-FAC). Children on HD had increased LVM and LVMI in addition to abnormal LV and RV diastolic function as demonstrated by higher LV/RV E/A and LV/RV E/E′, RVE/A values. The prevalence of LVH among children on HD was 80% (32 children). When compared to healthy children, children on HD showed substantially increased 4D LV/RV volumes as well as reduced 4D LV/RV EF, 4D LV-GLS, RVEF, 4D TAPSE, and 4D FAC (indicating LV/RV systolic dysfunction).

**Table 4:** 2D echo-Doppler parameters in children receiving chronic hemodialysis and controls

Variables	Stage 5 CKD patients (*N* = 40)	Controls (*N* = 40)	*P*
IVS (mm)	8.4 ± 1.9	6.1 ± 0.8	0.0001
PW (mm)	8.6 ± 2.2	5.8 ± 0.8	0.0001
LVEDD (mm)	45.4 ± 7.6	34.9 ± 3.1	0.0001
LVESD (mm)	33.4 ± 6.1	22.4 ± 2.7	0.0001
LVEF-MM (%)	55.4 ± 4.8	68.8 ± 6.2	0.0001
LVM (gm)	125.8 ± 50.7	51.2 ± 12.1	0.0001
LVMI (g/m^2^)	146.0 ± 80.0	60.0 ± 18.9	0.0001
LVMI (g/m^2.7^)	88.5 ± 45.3	33.9 ± 12.5	0.0001
RWT	0.39 ± 0.1	0.33 ± 0.1	0.0001
MAPSE (mm)	9.9 ± 1.3	13.6 ± 1.4	0.0001
MV E/A	1.8 ± 0.6	1.1 ± 0.1	0.0001
LV-MPI	0.54 ± 0.2	0.27 ± 0.0	0.0001
LV S′ (cm/s)	6.1 ± 1.6	14.3 ± 1.4	0.0001
LVE/E′	25 ± 1.0	7 ± 0.0	0.0001
LVEDV (mL)	146.8 ± 27.9	64.0 ± 7.3	0.0001
LVESV (mL)	64.6 ± 131.1	22.1 ± 4.6	0.0001
2D EF	55.8 ± 4.8	65.3 ± 7.0	0.0001
RV E/A	1.3 ± 0.5	1.0 ± 0.1	0.003
TAPSE (mm)	12.9 ± 1.9	20.4 ± 2.3	0.0001
FAC (%)	33.9 ± 3.8	50.2 ± 4.3	0.0001
PHT (mmHg)	43.8 ± 9.2	16.0 ± 3.1	0.0001
RV-MPI	0.44 ± 0.1	0.26 ± 0.0	0.0001
RV S′ (cm/s)	7.5 ± 1.4	14.8 ± 1.5	0.0001
RV E/E′	13 ± 0.0	4 ± 0.0	0.0001

*CKD* chronic kidney disease, *IVS* interventricular septum thickness, *PW* posterior wall thickness, *LVEDD* left ventricular end-diastolic dimension, *LVESD* left ventricular end-systolic dimensions, *EF-MM* ejection fraction by M-mode, *LVM* Left ventricular mass, *LVMI* Left ventricular mass index, *RWT* relative wall thickness, *MAPSE* mitral annular plane systolic excursion, *LVMPI* left ventricular myocardial performance, *LVEDV* left ventricular end-diastolic volume, *LVESV* left ventricular end-systolic volume, *TAPSE* tricuspid annular plane systolic excursion, *FAC* fractional area change, *PHT* pulmonary hypertension, *RV-MPI* right ventricular myocardial performance index

**Table 5:** 4D echo-Doppler and speckle tracking parameters in children receiving chronic hemodialysis and controls

Variables	Stage 5 CKD patients (*N* = 40)	Controls (*N* = 40)	*P*
LV-GLS (%)	12.9 ± 2.0	23.9 ± 1.7	0.0001
RV-GLS	15.1 ± 2.4	28.1 ± 1.2	0.0001
4D LVEDV (mL)	143.5 ± 7.5	112.6 ± 14.8	0.0001
4D LVESV (mL)	59.6 ± 11.4	43.0 ± 8.3	0.0001
4D LVEF (%)	50.0 ± 2.6	58.2 ± 2.3	0.0001
4D LV-GLS (%)	12.1 ± 2.6	21.3 ± 2.3	0.0001
4D RVEDV (mL)	138.3 ± 9.0	112.2 ± 7.6	0.0001
4D RVESV (mL)	89.7 ± 8.1	30.3 ± 7.2	0.0001
4D RVEF (%)	38.5 ± 6.8	50.9 ± 2.5	0.0001
4D TAPSE (mm)	10.1 ± 1.8	17.4 ± 2.1	0.0001
4D LVEDV (mL)	143.5 ± 7.5	112.6 ± 14.8	0.0001

*CKD* chronic kidney disease, *LV-GLS* left ventricular global longitudinal shortening, *RV-GLS* right ventricular global longitudinal shortening, *LVEDV* left ventricular end-diastolic volume, *LVESV* left ventricular end-systolic volume, *LVEF* left ventricular ejection fraction, *RVEDV* right ventricular end-diastolic volume, *RVESV* right ventricular end-systolic volume

The correlations between the duration of HD therapy and Holter parameters, as well as 2D and 4D echocardiographic variables, are summarized in Supplementary Table 1. The duration of HD therapy showed a negative correlation with rMSSD and HF (markers of parasympathetic activity), LV mass, LVMI, 2D/4D LV-EF, and RV-systolic function (RV-GLS, RV-EF, and 2D/4D TAPSE). In contrast, LF/HF (a marker of the balance of ANS function) and 4D LV volumes were positively correlated with the duration of HD therapy.

Supplementary Table 2 shows the correlations between LV/RV GLS and TAPSE with different Holter parameters. The results showed a positive correlation between 2D/4D LV shortening, indices of parasympathetic activity (pNN50 and rMSSD), and ANS balance (LF/HF).

LVMI correlated negatively with MAPSE (*r* = − 0.462, *p* < 0.0001), LV-GLS (*r* = − 0.580, *p* < 0.0001), TAPSE (*r* = − 0.502, *p* < 0.0001), RV-GLS (*r* = 0.572, *p* < 0.0001), but correlated positively with LV/RV E/E’ (*r* = 0.516, 0.491; *p* < 0.0001, respectively).

### ROC curve to detect the 4D-LV and RV cutoff values to identify systolic dysfunction among the studied children on HD

**Fig. 2:**
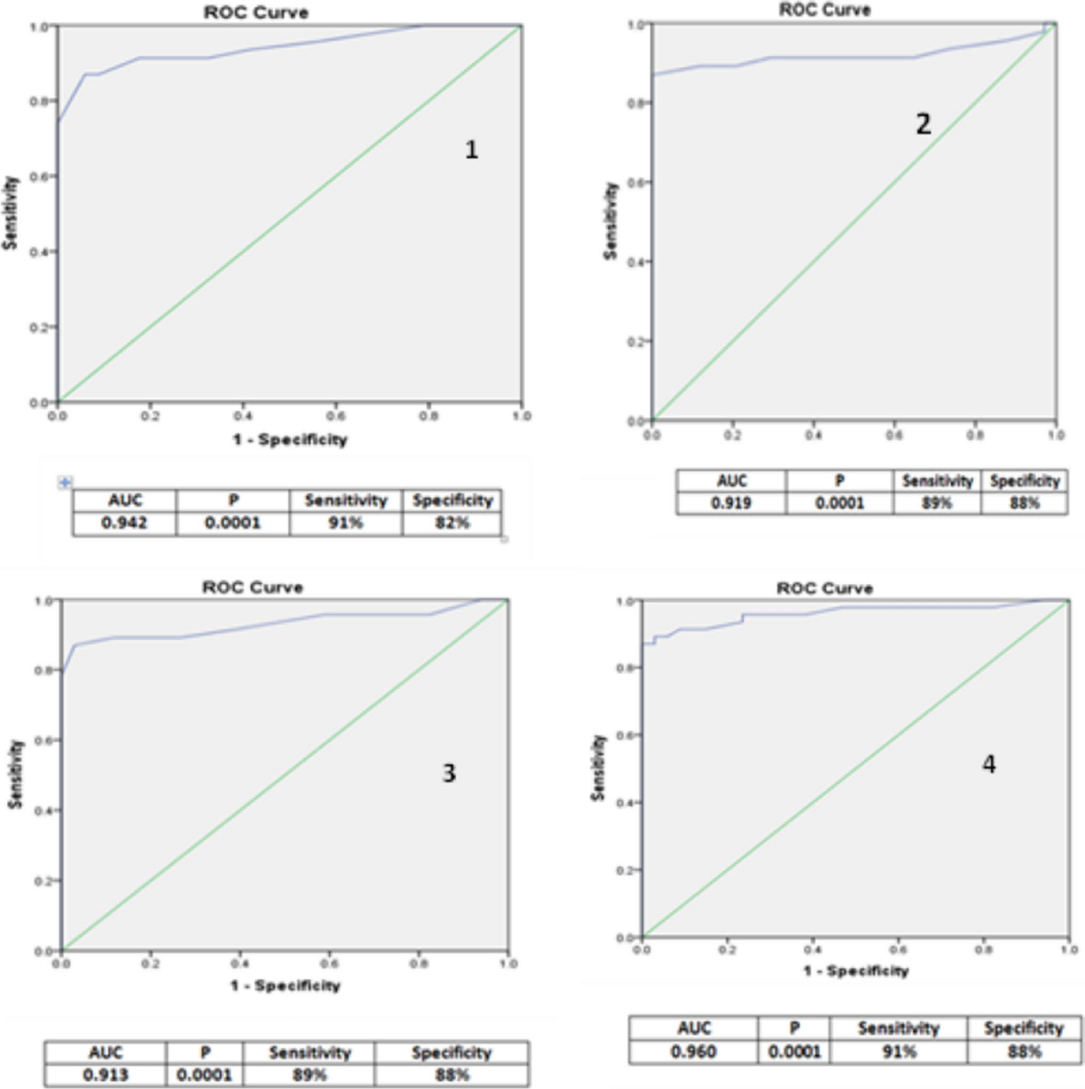
Receiver operating characteristic (ROC) curve to determine cutoff value for 4D systolic dysfunction among children on chronic hemodialysis. (1) 4D LVEF, (2) 4D LVGLS, (3) 4D TAPSE, (4) 4D RV-EF, and (5) 4D RV FAC. Abbreviations: LVEF, left ventricular ejection fraction; LV-GLS, LV global longitudinal shortening; TAPSE, tricuspid annular plane systolic excursion

A ROC curve was constructed to identify the cutoff value for 4D LV and RV systolic dysfunction in children on HD. The cutoff point for LV systolic dysfunction was a 4D LVEF of 52.5% (sensitivity = 91%, specificity = 82%, and AUC = 0.942). Figure 2 shows that the cutoff value to identify LV systolic dysfunction was 4D LV-GLS = 9.5%, with corresponding sensitivity, specificity, and AUC values of 89%, 88%, and 0.919.

For RV systolic dysfunction, a cutoff of 12.5 mm was established using 4D TAPSE (sensitivity = 89%, specificity = 88%, and AUC = 0.913). Additionally, the value for detecting RV systolic dysfunction was a 4D RV-EF of 42.9% (sensitivity = 91%, specificity = 88%, and AUC = 0.960). Furthermore, the 4D RV-FAC of 36.5% could serve as a cutoff point (sensitivity = 89%, specificity = 85%, and AUC = 0.950) (Fig. 2).

### Mortality and morbidity among children on HD in our study

**Fig. 3:**
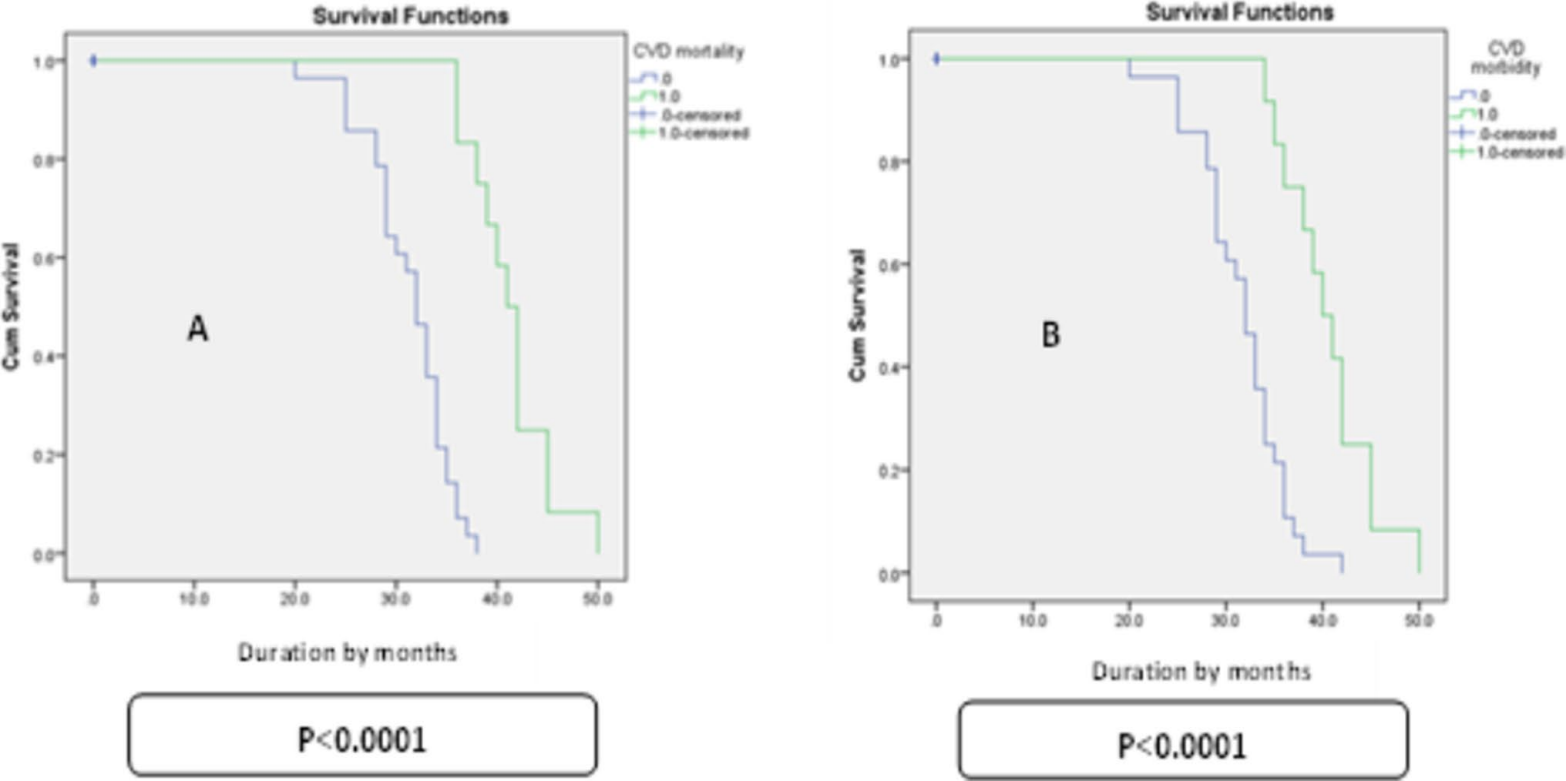
Kaplan–Meier curves depicting all-cause mortality (**A**) and all-cause morbidity (**B**) among children on chronic hemodialysis

Supplementary Table 3 and Fig. 3 display all-cause mortality and morbidity rates among the included children on HD. Over a follow-up period of 34.5 ± 16.8 months, the 3-year overall mortality rate was 10 (25%) patients, and the 3-year overall morbidity rate was 15 (37.5%) patients. Sudden cardiac death was the primary cause of death among the enrolled patients (5 patients), followed by cerebral bleeding/thrombosis (3 patients), and recurrent respiratory infection (2 patients).

Higher mortality among children on HD was identified to be independently correlated with reduced RV-GLS (hazard ratio [HR], 1.378; 95% CI, 1.227–1.547; *p* < 0.0001), lower rMSSD value (HR, 1.114; 95% CI, 1.007–1.232; *p* < 0.038), and impaired LV-GLS (HR, 2.015; 95% CI, 1.596–2.545; *p* < 0.0001).

## Discussion

In the current prospective study, we investigated the relationship between autonomic imbalance and ventricular dysfunction among children on HD and concluded that (1) both 2D/4D echocardiographic left and right ventricular myocardial deformation parameters were primarily reduced in children on HD in addition to impairment of left and right ventricular systolic function evaluated by conventional echo-Doppler parameters, (2) reduced vagal activity resulting in unopposed sympathetic activity suggesting cardiac autonomic dysfunction as seen by impaired time and frequency domain, (3) left and right ventricular myocardial deformation were closely correlated with different Holter parameters, (4) the cutoff values of 4D left and right ventricular systolic dysfunction using different 4D echocardiographic parameters could be defined using ROC, and (5) mortality was independently correlated with impaired vagal activity and reduced both left and right ventricular myocardial deformation.

Hemodialysis is recognized as a stressful condition for cardiac functions [17]. While cardiovascular mortality in patients on HD is still greater than in the general population [23], there is not enough information available to determine how acute HD affects children’s cardiac function and cardiac autonomic function, which requires more investigations. In the current study, CKD children after HD sessions showed significant impairment of 2D LV and RV systolic function, evident by lower LVEF (despite the values still being within the normal range), mitral and tricuspid plane systolic excursion, LV/RV-global systolic shortening, and RV-FAC, in addition to a higher LV/RV myocardial performance index compared to controls. Moreover, children on HD had significant impairment of 4D LV/RV-EF, 4D LV-global longitudinal shortening, and 4D RV systolic function (both tricuspid plane systolic excursion and 4D RV-FAC) compared to healthy children. According to Voigt et al. [24], 2D STE is thought to be a more sensitive technique for detecting subclinical global and segmental ventricular functional alterations, offering additional information than conventional measurements. Following HD sessions, Rakha et al. [15] observed a significant drop in LV-GLS. Despite normal LVEF values, LV-GLS was the only index of systolic function that showed intradialytic deterioration. This suggests that more advanced non-invasive imaging methods might be essential to identify the deleterious effects of HD in children, which likely occur repeatedly with every HD session [25]. Accordingly, Elshamaa et al. [13] reported that LV-GLS significantly reduced in pediatric HD patients with a further reduction after each HD session.

Predisposition to myocardial ischemia and stiffness is higher in the subepicardium than in the subendocardium. According to Elshamaa et al. [13], cardiac stiffness may be a contributing factor to alterations in the function of the LV long axis in patients with CKD earlier than the function of the LV short axis. Moreover, it was noted that within a year of starting dialysis, HD patients with reversible segmental LV dysfunction may develop fixed segmental systolic dysfunction, which would lower global LV function and impair hemodynamics during further dialysis treatments [26]. Consequently, myocardial stunning is thought to be cumulative with subsequent ischemic episodes and is strongly linked to a higher risk of cardiovascular events and death [27].

The current study demonstrates that children on HD have impaired LV and RV diastolic function, confirmed by higher LV/RV E/A and LV/RV E/E′ ratios relative to controls. According to Mostafa et al. [28], diastolic dysfunction usually manifests prior to systolic dysfunction in pediatric CKD. Diastolic dysfunction may be associated with volume status, reduced LV compliance, an inflammatory response caused by uremic toxins, or the maladaptive hypertrophic response in these patients [28].

In addition to dialysis treatment, volume control is mainly accomplished by reducing fluid consumption, which can be challenging for all dialysis patients [29]. According to available data, aggressive fluid removal as well as hypervolemia might cause circulatory stress. Targeting euvolemia using conventional hemodialysis therapy may be detrimental in the absence of reliable volume-measuring instruments [30]. Optimal weight is the lowest weight a patient can tolerate without experiencing hypotension or apparent fluid overload [31]. The conventional method of determining optimal weight, often known as “dry weight,” involves a clinical evaluation that considers blood pressure trends, weight fluctuations, and the presence of clinical symptoms of volume overload [32]. High ultrafiltration requirements, hypertension, LV hypertrophy, and decreased urine output were all correlated with severe anemia as risk factors for fluid overload symptoms [33]. This shows that rather than having a compromised erythropoietic response, some dialysis patients with anemia may have low hemoglobin levels because of red cell mass dilution induced by volume overload. The association between volume overload and low hemoglobin concentration in patients with treatment-resistant anemia can be better understood by paying close attention to volume status and “challenging” dry weight with enhanced ultrafiltration [34]. Anemia in children with CKD has been linked to adverse cardiovascular events and LV remodeling [35]. Moreover, in patients with CKD, elevated serum phosphate levels have detrimental effects that include the development and progression of secondary hyperparathyroidism and extravascular metastatic calcification, which increases the risk of cardiovascular morbidity and mortality [36].

In our study, children on HD showed impaired HRV parameters as evidenced by lower LF, HF, pNN50, rMSSD, and SDNN compared to healthy children. Additionally, those children on HD exhibited significantly higher heart rates and were more likely to develop atrial and ventricular arrhythmias. It is known that the ANS plays a key role in maintaining hemodynamic stability. The observed uremic cardiac autonomic dysfunction in patients undergoing chronic HD is characterized by sympathetic hyperactivity and parasympathetic deterioration [37]. In agreement with our results, Salman [38] reported that among patients with CKD, autonomic dysfunction is a leading cause of cardiovascular morbidity and mortality. In these patients, an increased sympathetic activity and a reduction in parasympathetic tone were observed. Altered HRV, manifestations of cardiac autonomic dysfunction, may lead to an increased risk of developing fatal arrhythmias and SCD [39]. Overproduction of inflammatory mediators was associated with activation of the vagal inflammatory reflex in CKD patients. Despite this, kidney replacement therapies fail to adequately restore the ANS to a balanced state. Currently, the only ways to restore the sympathovagal imbalance are nephrectomy and renal denervation [39]. Hyperactivation of the sympathetic system not only leads to an increased basal heart rate (as observed in our study), but also promotes myocardial hypertrophy and fibrosis, which are associated with an increased risk of SCD [40].

Among our studied population, the duration of HD therapy negatively correlated with both LV and RV systolic function (identified by 2D LV/RV-EF, LV/RV-GLS, 2D/4D TAPSE), LVM/LVMI, and markers of parasympathetic tone (evident by HF and rMSSD). On the other hand, the duration of HD therapy positively correlated with LVESD, 4D LV volumes, and LF/HF as an index of ANS balance. Contradictory to our results, Rakha et al. [15] did not find a relationship between the duration of HD therapy with LV-GLS, or EF. However, Do Val et al. [41] confirmed the effect of dialysis duration on LVM among children with CKD. In a different study, which included 64 dialysis patients, there was no correlation between LVM and the duration of dialysis [42].

We could define the cutoff values of 4D LV-EF, 4D LV-GLS, 4D TAPSE, 4D RV-EF, and 4D FAC among HD children (52.5%, 9.5%, 12.5 mm, 42.9%, and 36.5%, respectively) to identify 4D LV and RV systolic dysfunction in HD children. To the best of our knowledge, the current study is the first to define the cutoff value for both 4D LV and RV systolic dysfunction among CKD children on regular HD. Charfeddine et al. [43] found that 4D LV end-diastolic and systolic volume index and shortening decreased remarkably after HD.

The 3-year overall mortality and morbidity rates were 25% and 37.5% among our studied patients, with cardiovascular causes being the primary detectable reasons for both mortality and morbidity. Impaired LV-GLS and RV-GLS, along with lower rMSSD (a marker of impaired vagal tone), were independent predictors for higher mortality among the included children on HD. Liu et al. [44] demonstrated that LV-GLS is a prognostic predictor of all-cause mortality in stable adult HD patients with preserved LVEF. Increased cardiovascular morbidity and mortality have been documented by several publications in individuals with renal insufficiency, particularly in those undergoing maintenance hemodialysis. According to Rajaa et al. [45], cardiovascular events account for over half of the deaths that occur in dialysis patients. According to Msaad et al. [46], Mpio et al. [47], and Lahrach et al. [48], the average patient mortality rate in patients on HD was 22.7% due to cardiovascular reasons. According to Jadoul et al. [49], patients on HD have a relatively high incidence of SCD. According to Makar and Pun [50] and Tereshchenko et al. [51], SCD is responsible for two-thirds of all cardiac deaths and one-fourth of all deaths.

## Strengths and limitations

The strength of the current study is that it could identify autonomic cardiovascular profiles. Shortening imaging and 4D echocardiography are essential for providing valuable information for the assessment of cardiovascular risk in children with CKD on regular HD. However, we had several limitations. First, as this was a small-sample, single-center preliminary investigation, more extensive and large-sample prospective research is required to validate the study’s conclusions. Second, since the echocardiographer who obtained the images was aware of the dialysis time, blinding the study was not feasible. Thirdly, in comparison to 2D STE, the temporal and spatial resolutions of the 4D STE are comparatively lower. Several patients were excluded due to inadequate images that were unsuitable for STE analysis and inadequate ECG signal recordings that interfered with the acquisition of echocardiographic data. Finally, we did not address dialysis adequacy and uremia complications, such as secondary hyperparathyroidism, which might also be possible confounders.

## Conclusions

Children with CKD undergoing HD showed impaired myocardial deformation, as evidenced by 2D/4D echo-Doppler indices. These patients also exhibited cardiac autonomic dysfunction characterized by increased sympathetic activity and decreased vagal activity. In children on HD, myocardial deformation indices were closely correlated with parameters of cardiac autonomic function. Mortality among children on HD was independently correlated with reduced left and right ventricular global longitudinal shortening as well as lower rMSSD. Our findings provide support for the implementation of comprehensive echocardiographic assessment methods and cardiac autonomic function evaluation to identify high-risk groups among children on HD.

## Supplementary Information

Below is the link to the electronic supplementary material.

Graphical abstract (PPTX 162 KB)

Supplementary file2 (DOCX 21 KB)

## Data Availability

The datasets generated during and/or analyzed during the current study are available from the corresponding author on reasonable request.
