# Supplementary material for: The association between systolic and diastolic dysfunction and autonomic nervous system function in children receiving chronic hemodialysis
Source: Pediatr Nephrol. 2025 Jan 28;40(8):2599–610. doi: 10.1007/s00467-024-06577-1 (PMC12187883; doi:10.1007/s00467-024-06577-1)
Supplement: Supplementary file 1 — Graphical abstract (PPTX 162 KB) [file 467_2024_6577_MOESM1_ESM.pptx]

## Slide 1
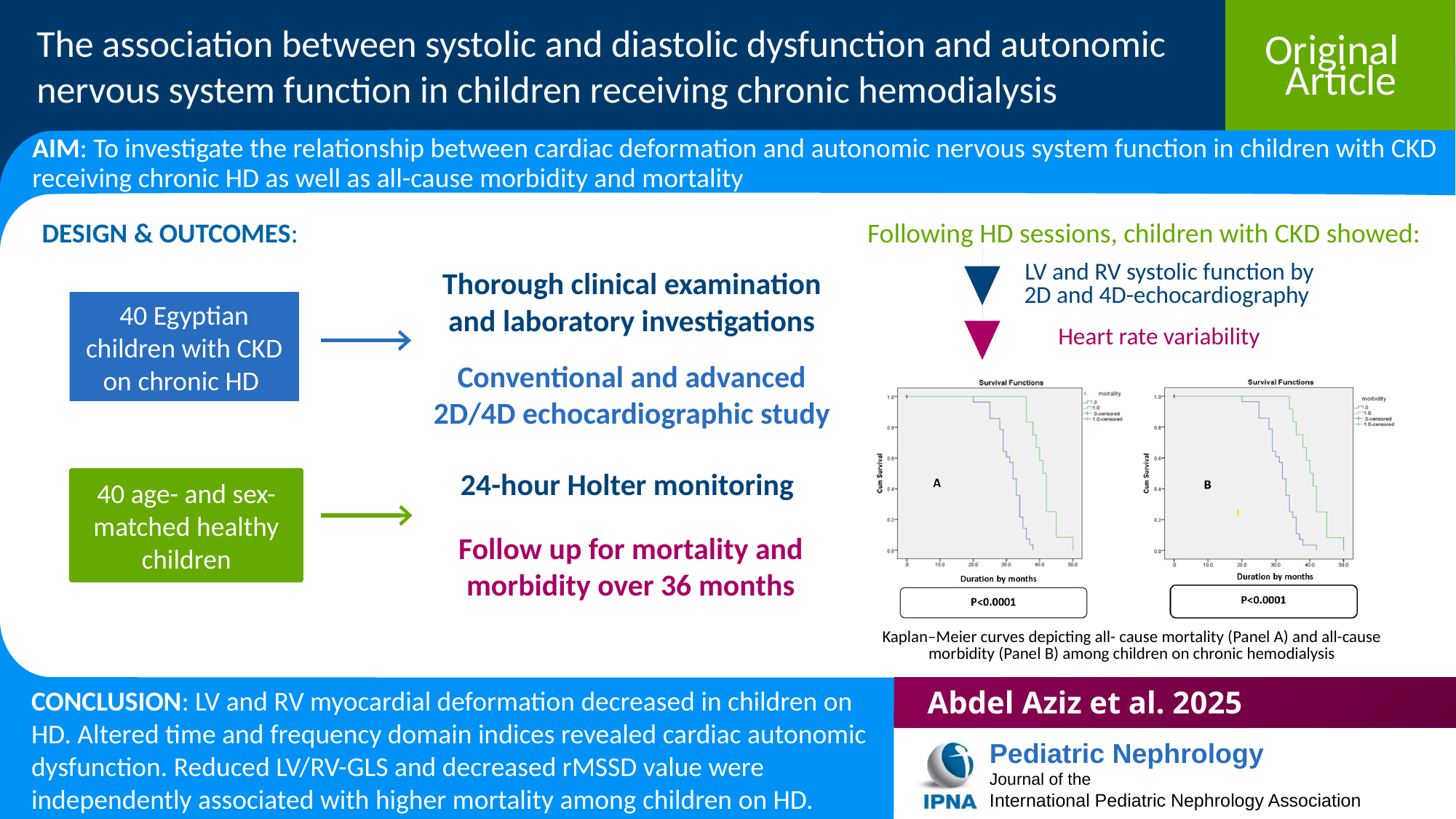

The association between systolic and diastolic dysfunction and autonomic nervous system function in children receiving chronic hemodialysis
AIM: To investigate the relationship between cardiac deformation and autonomic nervous system function in children with CKD receiving chronic HD as well as all-cause morbidity and mortality
DESIGN & OUTCOMES:
Following HD sessions, children with CKD showed:
LV and RV systolic function by 2D and 4D-echocardiography
Thorough clinical examination and laboratory investigations
40 Egyptian children with CKD on chronic HD
Heart rate variability
Conventional and advanced 2D/4D echocardiographic study
24-hour Holter monitoring
40 age- and sex- matched healthy children
Follow up for mortality and morbidity over 36 months
Kaplan–Meier curves depicting all- cause mortality (Panel A) and all-cause morbidity (Panel B) among children on chronic hemodialysis
Abdel Aziz et al. 2025
CONCLUSION: LV and RV myocardial deformation decreased in children on HD. Altered time and frequency domain indices revealed cardiac autonomic dysfunction. Reduced LV/RV-GLS and decreased rMSSD value were independently associated with higher mortality among children on HD.
