# Supplementary material for: The association between systolic and diastolic dysfunction and autonomic nervous system function in children receiving chronic hemodialysis
Source: Pediatr Nephrol. 2025 Jan 28;40(8):2599–610. doi: 10.1007/s00467-024-06577-1 (PMC12187883; doi:10.1007/s00467-024-06577-1)
Supplement: Supplementary file 2 — Supplementary file2 (DOCX 21 KB) [file 467_2024_6577_MOESM2_ESM.docx]

**Supplementary Table 1.** Correlations between duration of HD therapy with both 2D/4D parameters and Holter parameters

| Duration of dialysis vs. | r | P |
| --- | --- | --- |
| LVESV (mL) | 0.427 | 0.01 |
| EF-MM (%) | -0.365 | 0.02 |
| 2D-EF (%) | -0.3939 | 0.01 |
| LVM (gm) | -0.461 | 0.003 |
| LVMI (gm/m^2.7^) | -0.383 | 0.02 |
| LV-GLS (%) | -0.874 | 0.0001 |
| 4D LVEDV (mL) | 0.452 | 0.003 |
| 4D LVESV (mL) | 0.517 | 0.001 |
| 4D LVEF (%) | -0.325 | 0.04 |
| 4D LVGLS (%) | -0.849 | 0.0001 |
| TAPSE (mm) | -0.842 | 0.0001 |
| RV-GLS (%) | -0.833 | 0.0001 |
| 4D RVEF (%) | -0.430 | 0.01 |
| 4D TAPSE (mm) | -0.799 | 0.0001 |
| HF (nu) | -0.431 | 0.01 |
| LF/HF | 0.452 | 0.003 |
| rMSSD (ms) | -0.639 | 0.0001 |

**Abbreviations:** *HD*, hemodialysis; *LVESV*, left ventricular end systolic volume; *EF-MM*, ejection fraction by M-mode; *LVM*, left ventricular mass; *LVMI*, left ventricular mass index; *LV-GLS*, LV global longitudinal strain; *LVEDV*, LV end diastolic volume; *TAPSE*, tricuspid annular plane systolic excursion; *RV-GLS*, right ventricular global longitudinal strain; *HF*, high frequency; *LF*, low frequency; *rMSSD*, root mean square of the difference between successive normal intervals.

**Supplementary Table 2.** Correlations between Holter parameters with LV, RV 2D/4D global shortening and TAPSE.

| 1. 2D LV-GLS vs. | r | P |
| --- | --- | --- |
| LF/HF | 0.927 | 0.0001 |
| LF (nu) | 0.846 | 0.0001 |
| HF (nu) | -0.921 | 0.0001 |
| pNN50 (%) | 0.933 | 0.0001 |
| rMSSD (ms) | 0.944 | 0.0001 |
| 1. 4D LV-GLS vs. |  |  |
| LF/HF | 0.949 | 0.0001 |
| LF (nu) | 0.888 | 0.0001 |
| HF (nu) | -0.952 | 0.0001 |
| pNN50 (%) | 0.962 | 0.0001 |
| rMSSD (ms) | 0.977 | 0.0001 |
| 1. 2D RV-GLS vs. |  |  |
| LF/HF | 0.909 | 0.0001 |
| LF (nu) | 0.848 | 0.0001 |
| HF (nu) | -0.913 | 0.0001 |
| pNN50 (%) | 0.908 | 0.0001 |
| rMSSD (ms) | 0.931 | 0.0001 |
| 1. 4D TAPSE |  |  |
| LF/HF | 0.942 | 0.0001 |
| LF (nu) | 0.883 | 0.0001 |
| HF (nu) | -0.948 | 0.0001 |
| pNN50 (%) | 0.956 | 0.0001 |
| rMSSD (ms) | 0.984 | 0.0001 |

**Abbreviations:** *LV-GLS*, left ventricular global longitudinal shortening; *LF*, low frequency; *HF*, high frequency; *pNN50*, percentage of differences>50 ms between successive normal RR intervals; *rMSSD*, root mean square of the difference between successive normal intervals; *RV-GLS*, right ventricular longitudinal shortening; *TAPSE*, tricuspid annular plane systolic excursion.

**Supplementary Table 3.** Primary and secondary endpoints among HD children.

| Variables | N=40 |
| --- | --- |
| All-cause mortality:   1. Sudden cardiac death 2. Cerebral haemorrhage/thrombosis 3. Recurrent respiratory infection | **10 (25%)**  5 (12.5%)  3 (7.5%)  2 (5%) |
| All-cause morbidity   - Significant arrhythmias - Repeated infections - Vascular access complications - Cerebrovascular stroke | **15 (37.5%)**  5 (12.5%)  5 (12.5%)  3 (7.5%)  2 (5%) |

**Abbreviations:** *HD*, hemodialysis.
